# Comparison of the Determinants of the “Chronic Obstructive Pulmonary Disease Assessment Test” (CAT) and the “Asthma Control Test” (ACT) in Patients with Asthma–COPD Overlap

**DOI:** 10.3390/jcm13216367

**Published:** 2024-10-24

**Authors:** Cristina Aljama, Galo Granados, Francisco Javier Callejas-González, Carlos Martínez-Rivera, Abel Pallarés-Sanmartín, Laura Rodríguez-Pons, Eva Cabrera-César, Eduardo Márquez-Martín, Ana Boldova-Loscertales, Elsa Naval-Sendra, Beatriz Abascal-Bolado, Carlos Cabrera-López, Marc Miravitlles, Cristina Esquinas, Miriam Barrecheguren

**Affiliations:** 1 Pneumology Department, Hospital Universitari Vall d’Hebron/Vall d’Hebron Institut de Recerca (VHIR), Vall d’Hebron Barcelona Hospital Campus, 08035 Barcelona, Spain; cris.aljama94@gmail.com (C.A.); mdgdgr85@gmail.com (G.G.); crise4@hotmail.com (C.E.); miriam.barrecheguren@vallhebron.cat (M.B.); 2 Pneumology Department, Complejo Hospitalario Universitario de Albacete, 02008 Albacete, Spain; f.javiercallejas@hotmail.com; 3 Pneumology Department, Hospital Germans Trias i Pujol, 08916 Badalona, Spain; carlosmartinezrivera.cmr@gmail.com (C.M.-R.); laurarodriguezpons@gmail.com (L.R.-P.); 4 Pneumology Department, Complejo Hospitalario Universitario de Ourense, 32005 Ourense, Spain; apallare@hotmail.com; 5 Pneumology Department, Hospital Universitario Virgen de la Victoria, 29010 Málaga, Spain; evacabreracesar@gmail.com; 6 Pneumology Department, Hospital Virgen del Rocío, 41013 Sevilla, Spain; eduardomarquezmartin@gmail.com; 7 Pneumology Department, Hospital Royo Villanova, 50015 Zaragoza, Spain; anabl81@yahoo.es; 8 Pneumology Department, Hospital Universitario de La Ribera, 46600 Alzira, Spain; elsa.navals@gmail.com; 9 Pneumology Department, Hospital Universitario Marqués de Valdecilla, 39008 Santander, Spain; b_abascal@hotmail.es; 10 Pneumology Department, Hospital Universitario Dr. Negrín, 35010 Las Palmas de Gran Canaria, Spain; ccablopn@gmail.com

**Keywords:** CAT, ACT, asthma–COPD overlap, quality of life

## Abstract

**Objective:** The objective of this study was to investigate which of two short questionnaires, the Asthma Control Test (ACT) or the COPD Assessment Test (CAT), correlates better with severity variables and whether they share similar determinants in patients with asthma–COPD overlap. **Method:** This observational, cross-sectional, multicentric study included smokers and former smokers of more than 10 pack-years, with non-fully reversible airflow obstruction and either a concomitant diagnosis of asthma or signs of type 2 inflammation, from 15 centres in Spain. **Results:** A total of 157 patients were included, 109 (69.4%) were men, the mean age was 63.3 (SD: 9) years and the mean FEV1 (%) was 59.7% (SD: 20.5%). The mean CAT score was 14.5 (SD: 8.7), and the mean ACT score was 17.9 (SD: 5.2). Both scores showed good correlations (r = 0.717; *p* < 0.001). In the multivariate analysis, the Hospital Anxiety and Depression Scale and mMRC dyspnoea scores were independently and significantly associated with both the CAT and ACT scores; however, age was only significantly associated with the CAT, and the EQ-5D scores and the number of exacerbations in the previous year were only significantly associated with the ACT scores. The ACT had a slightly better predictive value for exacerbations than the CAT (AUC = 0.70 (95% CI: 0.62 to 0.79 vs. 0.65 (95% CI: 0.56 to 0.74))). **Conclusions:** There is a good correlation between ACT and CAT scores in patients with ACO. However, severe patients scored worse on the CAT than the ACT. Anxiety, depression and dyspnoea were significantly associated with both the CAT and ACT scores. The ACT was a slightly better predictor of exacerbations than the CAT in this population.

## 1. Introduction

Chronic obstructive pulmonary disease (COPD) and asthma are two very prevalent diseases in the adult population. In Spain, the estimated prevalence of COPD is around 11.8% of the total population over 40 years of age [1], while the prevalence of asthma in adulthood is estimated to be 5.1% [2]. Both diseases are characterised by being chronic inflammatory diseases that affect the airway, with airflow obstruction that is usually more variable in asthma and not fully reversible in COPD. Due to the high prevalence of both diseases, they may coexist in asthmatics who smoke and develop emphysema and chronic bronchitis or smokers with COPD who have a predominant T2 type of inflammation [3,4,5]. This T2 inflammation in COPD is characterised by peripheral blood and tissue eosinophilia, high fractional exhaled nitric oxide (FeNO) levels, high bronchial hyperresponsiveness and better responses to treatment with oral and inhaled corticosteroids (ICSs) [5,6,7,8]. These two types of COPD patients, those with coexisting asthma and those with predominant T2 COPD, were grouped together and defined as asthma–COPD overlap (ACO) by the Spanish COPD treatment guidelines because of their similar responses to ICSs [9]. More recently, other guidelines have adopted similar definitions of ACO [10,11,12]. Among the population of patients with COPD, approximately 25% to 30% fulfil the definition of ACO [13,14,15], and these individuals usually have more exacerbations and hospitalisations and a worse quality of life compared with persons with asthma or COPD alone [7,13,16,17,18,19]. However, no patient-reported outcomes have been developed or their performances specifically compared in this population.

The questionnaire most widely used for COPD in clinical practice is the COPD Assessment Test (CAT) [20], which has been adopted by the GOLD [21] and other guidelines [22] for managing initial pharmacological treatment in COPD. On the other hand, the Asthma Control Test (ACT) [23] is extensively used to determine the degree of clinical control of asthma. It is not clear if these two short questionnaires are similarly valid in patients with ACO or whether they measure different dimensions of the disease and have different determinants in this population.

The objectives of our study were: (1) to determine if there is a correlation between the scores of the ACT and CAT questionnaires in patients with ACO; (2) to identify the determinants of CAT and ACT scores; (3) to analyse the correlations between ACT and CAT scores and severity variables (i.e., exacerbations, lung function, exercise capacity and dyspnoea) in patients with ACO and (4) to establish the most appropriate ACT or CAT cut-off point to predict exacerbations in these patients.

## 2. Materials and Methods

### 2.1. Design of This Study

This was an observational, cross-sectional, multicentric study performed in the pulmonology outpatient clinics of 15 centres in Spain from January 2019 to January 2020. This study was approved by the Ethics Committee of the University Hospital of Albacete (Spain), number 11/2017, as the coordinating centre and by all the other participating centres. All the patients provided signed informed consent to participate in this study.

### 2.2. Participants

Patients fulfilling the diagnostic criteria of ACO according to the Spanish GesEPOC-GEMA consensus were consecutively included in this study [9]. The patients fulfilling the COPD criteria were over 40 years of age, smokers or ex-smokers with cumulative consumptions of at least 10 pack-years, with post-bronchodilator spirometry showing a forced expiratory volume in 1 s (FEV1)/forced vital capacity (FVC) < 0.7 and either (a) a diagnosis of asthma or (b) a very positive bronchodilator test (>15% and a 400 mL increase in FEV1) and/or peripheral blood eosinophilia > 300 cells/µL in the previous year [9]. Furthermore, the patients had to be clinically stable for at least four weeks since the resolution of the last exacerbation and not present other chronic respiratory diseases such as lung cancer, the sequelae of pulmonary tuberculosis, the presence of clinically significant bronchiectasis or interstitial lung disease. The presence of severe comorbidities that could have interfered with the objectives of this study, i.e., severe cardiac, renal or liver insufficiency or severe cognitive impairment, was an exclusion criterion.

### 2.3. Measurements

The anthropometric and sociodemographic data of the patients were collected, as well as the number of exacerbations in the previous year, including the numbers of emergency room visits, hospital admissions and exacerbations that required corticosteroid and/or antibiotic treatment. The degree of dyspnoea was evaluated with the modified Medical Research Council (mMRC) scale [24]. Comorbidities were evaluated with the Charlson Index [25] and the COTE Index [26]. The disease severity was also calculated with the BODEx index [27]. To assess the levels of patient anxiety and depression, the Hospital Anxiety and Depression Scale (HADS) questionnaire was administered [28], and to measure quality of life, the European Quality of Life—5 Dimensions (EQ-5D) questionnaire was used [29,30]. The patients performed forced spirometry with a bronchodilator test and a six-minute walking test; according to international standards, only one measurement was requested [31]. Analytical data (C-reactive protein (CRP) values and fibrinogen and eosinophil count) were collected.

The CAT and ACT scores were obtained for each patient. The CAT is a validated, short, self-administered questionnaire that measures the impact of the disease in patients with 8 questions. The score ranges from 0 to 40, with 40 being the worst possible health state and 0 the best [20]. The CAT has an excellent correlation with other quality of life questionnaires [32,33] and allows assessment of the impact of COPD on health status within only a few minutes. On the other hand, the ACT is a self-administered questionnaire that provides a numerical score to assess the control of asthma. It comprises five questions regarding aspects of asthma control relevant to patients. Each question is answered on a 5-point scale, with a total score ranging from 5 to 25; higher scores indicate improved asthma control [23].

A threshold of ≤ 10 has been established as an indicator of low impact of the disease in the case of the CAT [20], and a score > 19 indicates controlled disease in the case of the ACT [23]. Patients presenting with ACT < 20 and CAT > 10 or ACT > 20 and CAT < 10 were classified as “concordant” and those with ACT > 20 and CAT > 10 or ACT > 20 and CAT > 10 as “discordant”.

### 2.4. Statistical Analysis

The results are expressed as absolute numbers and their corresponding percentages for qualitative variables, as means and standard deviation (SD) for quantitative variables with normal distribution and as medians and 25th to 75th percentiles for quantitative variables with non-normal distribution.

The CAT and ACT scores were compared based on the characteristics of the patients using the Student *t*-test or ANOVA tests with the Bonferroni correction for multiple comparisons in the case of qualitative variables. The Spearman correlation test was performed to determine if there were correlations between the CAT and ACT scores and other variables of interest, such as exacerbations, the BODE and BODEx indexes, FEV1, dyspnoea according to the mMRC scale and metres walked in the 6-min walk test.

Subsequently, to respond to the rest of the objectives, the scores of both questionnaires were established as dependent variables, and two different multivariate linear regression models were built, including sociodemographic and clinical variables as independent factors. Finally, the receiver-operating characteristic (ROC) curves of the CAT and ACT scores and exacerbations were calculated and compared using DeLong’s test. The results were defined as statistically significant, with *p* < 0.05. All the analyses were performed using the SPSS statistical package, version 27.0 (IBM Analytics, Armonk, NY, USA).

## 3. Results

### 3.1. Study Population

A total of 157 patients were included, of whom 109 (69.4%) were men, with a mean age of 63.3 (SD: 9) years, and 47 (29.9%) were active smokers. The diagnostic criteria of the ACO of the studied population are described in Table 1.

The mean FEV1 was 59.7% (SD: 20.5%), and the mean BODEx index was 2.1 (SD: 1.8). The mean EQ-5D index was 0.8 (SD: 0.2), and the visual analogue scale (VAS) score was 61.9 (SD: 20.4). The total HADS score was 10.4 (SD: 8.5). The mean eosinophil count was 317 cells/μL (SD: 270), and the mean fibrinogen level was 392.3 mg/dL (SD: 573). Table 2 shows the rest of the descriptive characteristics of the total sample.

### 3.2. CAT and ACT Scores and Their Correlation

**Figure 1:**
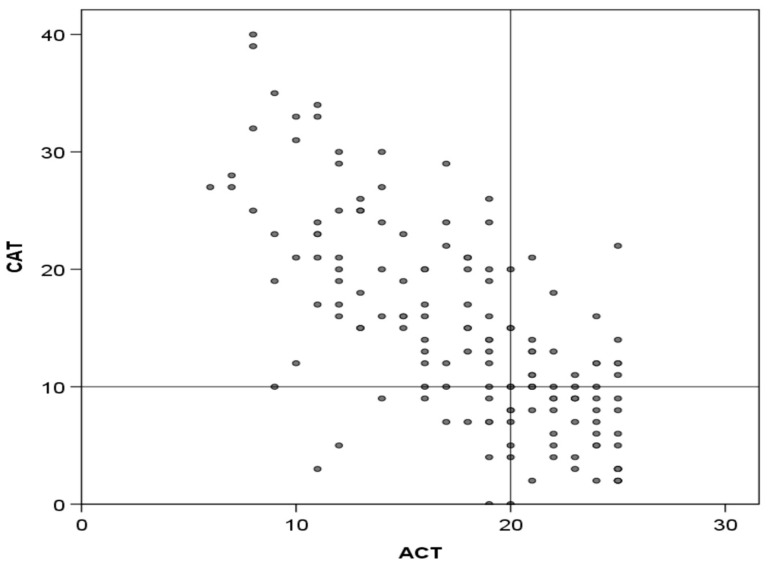
Correlation between CAT and ACT scores. Lines divide the area into those with good or poor CAT and ACT scores according to established cut-offs (<10 and >20, respectively). Correlation: Spearman, r = 0.717; *p* < 0.001.

The mean CAT score was 14.5 (SD: 8.7), while the mean ACT score was 17.9 (SD: 5.2). A total of 38 (24.2%) patients had CAT scores of <10, indicating a low impact, and 48 (30.5%) had ACT scores of >19, indicating good control. The scores of both questionnaires showed good correlation, with a Spearman r-value of 0.717 (*p* < 0.001) (Figure 1).

### 3.3. Correlations Between CAT and ACT Scores and Markers of Disease Severity

The variables that best correlated with the two questionnaires were the total HADS and HADS depression and anxiety scores, the mMRC dyspnoea scale scores, the EQ-5D index and VAS utility scores and the BODEx index scores. Their correlations with other variables, such as the number of exacerbations and lung function, were also significant, albeit poor (Table 3).

**Figure 2:**
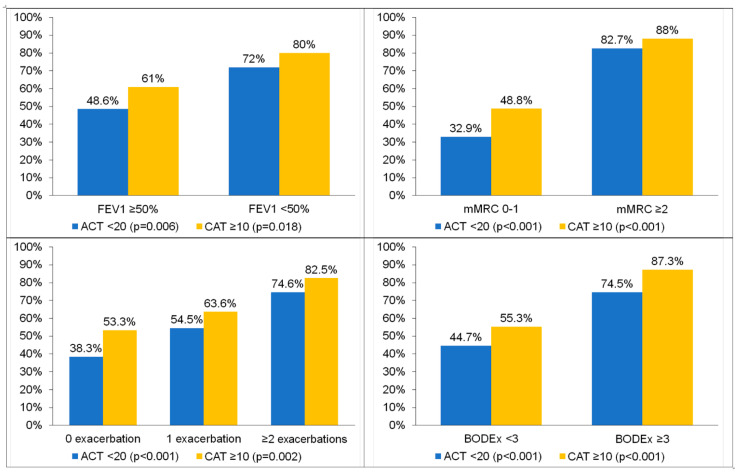
Percentages of patients with poor control (ACT < 20) or high impact of symptoms (CAT ≥ 10) according to different markers of severity of chronic respiratory disease. *p* = 0.006 and *p* = 0.018 for comparison between FEV1 < 50% and FEV ≥ 50% in the ACT and CAT, respectively. *p* < 0.001 and *p* < 0.001 for comparison between mMRC 0–1 and mMRC ≥ 2 in the ACT and CAT, respectively. *p* < 0.001 and *p* = 0.002 for comparison between 0 exacerbations, 1 exacerbation and ≥ 2 exacerbations in the ACT and CAT, respectively. *p* < 0.001 and *p* < 0.001 for comparison between BODEx < 3 and BODEx ≥ 3 in the ACT and CAT, respectively.

In general, among patients with greater disease severity, significantly more patients were classified as high-impact by the CAT than not controlled by the ACT. For example, among the patients with severe airflow obstruction (FEV1 predicted < 50%), 80% were classified as high-impact by the CAT and 72% were not controlled according to the ACT (*p* = 0.006). Similar results were obtained for the mMRC scale, the number of exacerbations in the previous year and the BODEx index (Figure 2).

### 3.4. Factors Significantly Associated with CAT and ACT Scores: Univariate and Multivariate Analyses

The variables significantly associated with the CAT and ACT scores differed somewhat. In the multivariate analysis, the HADS and the mMRC dyspnoea scores were independently and significantly associated with both the CAT and ACT scores; however, only age was significantly associated with the CAT and EQ-5D scores, while only the number of exacerbations within the previous year were significantly associated with ACT scores (Table 4).

### 3.5. Predictive Values of the CAT and ACT Scores for Exacerbations

**Figure 3:**
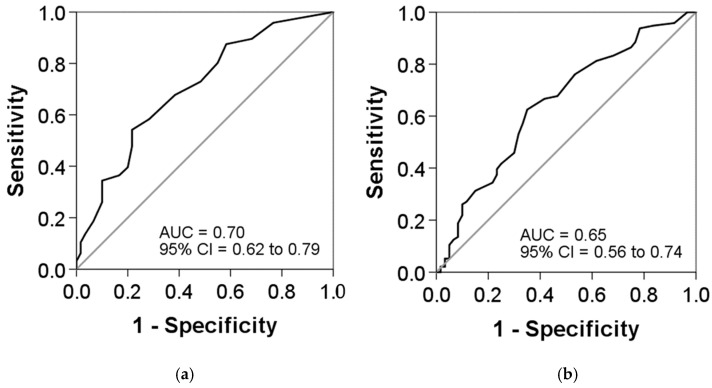
Receiver-operating characteristic (ROC) curves for the (**a**) ACT and (**b**) CAT scores as predictors of exacerbations: (**a**) area under the curve (AUC), 0.70; cut-off, 17.5; sensitivity, 54.2%; and specificity, 78.3% and (**b**) AUC, 0.65; cut-off, 12.5; sensitivity, 62.5%; and specificity, 65%. DeLong’s test indicated no significant difference between both AUCs (*p* = 0.351).

The ROC curves were used to investigate the predictive values of the CAT and ACT scores for exacerbations. The analyses showed a slightly better predictive value for the ACT, with an area under the ROC curve (AUC) of 0.70 (95% confidence interval [CI]: 0.62 to 0.79; *p* < 0.001), compared to the CAT questionnaire, with an AUC of 0.65 (95% CI: 0.56 to 0.74; *p* < 0.001), but a statistically significant difference was not achieved. The best cut-offs for the ACT and CAT questionnaires were 17.5 and 12.5, respectively (Figure 3).

### 3.6. Discordance Between the CAT and ACT Scores

The majority of patients were concordant in terms of either good control and low impact of symptoms (n = 40, 25.4%) or poor control and high impact (n = 78, 49.7%). However, the results were discordant in 39 (24.8%) subjects. In general, the concordant patients with good control and low levels of symptoms had better lung function, fewer exacerbations and better generic quality of life and anxiety and depression scores compared with the concordant patients with poor control and high symptom levels (Table 2).

We also evaluated whether the characteristics of the discordant patients with poor CAT and good ACT scores differed from the remaining discordant patients with good CAT and poor ACT scores. The results of this comparison showed that the patients with worse CAT but better ACT scores (n = 28, 71.8% of discordant) had significantly higher body mass indexes (BMIs), lower COTE comorbidity indexes and better FEV1/FVC ratios and also showed a trend towards younger age and better FEV1 scores (mL) compared with those with good CAT but poor ACT scores (Table 5).

## 4. Discussion

The results of the present study show good correlation between ACT and CAT scores in patients with ACO. However, almost 25% of patients were discordant, the majority of whom (78%) had good ACT but poor CAT scores. These patients had higher BMIs, fewer comorbidities and better FEV1/FVC ratios. Anxiety, depression and dyspnoea were significantly associated with both the CAT and ACT scores, but the CAT was also associated with age and the ACT with previous exacerbations and EQ-5D scores. Finally, the ACT was a slightly better predictor of exacerbations than the CAT in patients with ACO.

There is a need to develop patient-reported outcomes that can help clinicians to understand the impact of respiratory disease and even provide some guidance for treatment management. The ACT has shown to be useful in the assessment of asthma, both in exacerbations and in the stable state, as well as having a good relationship with the quality of life of patients with this disease [34]. In COPD, the CAT is a short questionnaire that correlates very well with Saint George’s Respiratory Questionnaire [33], which is considered the gold-standard questionnaire for specific health-related quality of life, and has been proposed by the GOLD strategy document, as well as by the Spanish COPD guidelines, as a criterion for initial pharmacological therapy for patients with COPD [22,35].

Although asthma and COPD have particular and differential characteristics, there is a group of patients that share characteristics of both diseases. The terminology to identify these subjects has long been debated, but irrespective of the name, there is general agreement that asthma and COPD may coexist, as stated in the last update of the GOLD document [21]. The current study was initiated just before the COVID-19 pandemic, and since it was conducted in Spain, we used the terminology of the Spanish consensus of 2017, which identified patients with criteria of both asthma and COPD or those with COPD and evidence of T2 inflammation as ACO [9]. The name and the criteria to define these patients can be challenged, but we believe that the validity of our results remains. The recommendation of GINA to call these patients “asthma + COPD” [35] is important because using only the term “COPD” or even ACO can hamper the prescription of highly effective drugs that are only approved for asthma [36]. However, for simplicity, in this article, the term ACO has been used, as this was used in the approved protocol.

In general, it was found that both questionnaires can be used for ACO patients, with similar reliabilities. There was a good correlation between the two questionnaires, and both had similar associations with the outcomes of interest, such as exacerbations, although the ACT performed somewhat better. Interestingly, despite the good correlation between the two questionnaires, almost one out of four patients were discordant, with the great majority (three quarters) showing good control in the ACT but high impact in the CAT. These individuals were younger and had more preserved lung function and fewer comorbidities compared to the remaining quarter, who had poor control in the ACT and low impact in the CAT.

These differences may, in part, be related to the different determinants of the scores of both tools. In the multivariate analysis, generic health-related quality of life and previous exacerbations were significantly and independently associated with ACT scores, whereas age was associated with CAT scores. More interestingly, however, was that both tools shared some determinants, such as the degree of dyspnoea and the level of anxiety and depression measured by the HADS questionnaire.

The association of ACT but not CAT scores with previous exacerbations may be related to the finding that the ACT scores were better determinants of the frequent exacerbator phenotype, with an AUC of 0.70, compared with 0.65 with the CAT. These differences are small, and the 95% CIs overlap, but they suggest that the ACT may be more determined by exacerbations than the CAT, perhaps due to the questions about the use of rescue treatment that are included in the ACT but not included in the CAT.

The significant association and the moderate and significant correlation observed between the HADS scores and both the ACT and CAT scores indicate the strong influence of mood on the scores of both questionnaires. Recent large studies have already shown this relationship. In another study in Spain, of 684 COPD patients, the level of depressive symptoms measured by the short Beck Depression Inventory explained 38% of the variance of the CAT scores [37], and a study of 3452 COPD patients from Eastern European countries observed that the clinical diagnosis of depression was the second most important variable significantly and independently associated with CAT scores, only after the degree of dyspnoea [38]. Our results suggest that psychological factors are also determinant in patients with ACO and may have a similar influence in the ACT and CAT scores.

We were also interested in comparing the classification of patients according to the established cut-off for poor asthma control (<20 units in the ACT) and a high level of COPD symptoms (>10 units in the CAT). Consistently, the CAT classified more participants as highly symptomatic compared to those poorly controlled as classified by the ACT. For example, among those with no exacerbations in the previous year, 53% had a high level of symptoms according to the CAT, but only 38% were not controlled, according to the ACT. Likewise, among patients with BODEx values of 3 or higher, 87.3% had high levels of symptoms according to the CAT, but only 74.5% were considered not controlled according to the ACT. These results indicate that the use of the CAT would result in a larger request for the intensification of treatment in ACO patients compared to the ACT.

Regarding treatment in patients with “asthma + COPD”, defined by similar criteria as in our population, Lommatzsch et al. [39] used the ACT to identify changes in disease control with the use of various biologics. So far, there is no biologic approved for use in COPD, but some of these products can be used in asthma, including asthma with some trends of COPD. The previously mentioned study included 20 asthmatics, smokers or former smokers of more than 10 pack-years and with FEV1/FVC values of <0.7 and observed significant improvements of a median of 7 units in the ACT scores with five different biologics; however, the authors did not use the CAT to evaluate symptoms in their patients. On the contrary, the CAT questionnaire was used in the METREO study, which investigated the efficacy of mepolizumab in patients with “type 2 COPD”, but the differences in the CAT scores between active treatment and placebo patients were not significant [40]. The future use of biologics in patients with “COPD and type 2 inflammation” or in “asthma + COPD” may require more information about the reliability and the prognostic values of both ACT and CAT in this group of patients.

The present study has several limitations. First, it was a cross-sectional study, with a single visit, without subsequent follow-up of the patients, especially in terms of symptoms and exacerbations. A follow-up of at least one year would allow observation of whether the correlation between the two tests is maintained over time and evaluate the prognostic value of the ACT and CAT scores for exacerbations. Another limitation is the use of a single definition of the ACO phenotype when including patients in this study. It is possible that if other definitions had been used, the patients included would have presented different baseline characteristics.

## 5. Conclusions

In patients with ACO, both the ACT and CAT symptom questionnaires showed similar results, although the ACT was more related to exacerbations and the CAT classified more patients as high-impact compared to the ACT. Both questionnaires have common determinants, such as dyspnoea, anxiety and depression. Longitudinal studies are needed to assess which of the two questionnaires is a better predictor of outcomes in these patients.

## Figures and Tables

**Table 1:** Diagnostic criteria of the ACO of the included population.

Diagnostic Criteria	N	%
Blood eosinophil count (BEC) > 300 cells/μL	61	38.9
Criteria of asthma	28	17.8
Criteria of asthma + BEC > 300 cells/μL	26	16.6
Bronchodilator test (BDT) > 15% and 400 mL	22	14
Criteria of asthma + BDT > 15% and 400 mL	12	7.6
BEC > 300 cells/μL + BDT > 15% and 400 mL	5	3.2
All three criteria	3	1.9

**Table 2:** Characteristics of the population of patients with ACO included in this study.

Variables	All (n = 157)	Concordant Poor Control, ACT < 20 and CAT > 10 (n = 78)	Concordant Good Control, ACT > 20 and CAT < 10 (n = 40)	Discordant (n = 39)	*p*-Value
Age, mean (SD)	63.3 (9)	61.9 (8.7)	63.9 (8.1)	65.5 (10.2)	0.041
Sex (male), n (%)	109 (69.4)	51 (65.4)	28 (70)	30 (77)	0.622
Smoker, n (%) Ex-smoker, n (%)	47 (29.9) 110 (70)	25 (32) 53 (67)	11 (27.5) 29 (72.5)	11 (28.2) 28 (71.8)	0.952
Smoking pack-years, mean (SD)	55.4 (154.5)	66 (218)	48.1 (24.9)	41.7 (24.7)	0.416
BMI, mean (SD)	27.7 (5.4)	28.4 (5.9)	26.9 (4.3)	27 (5.1)	0.115
Total Charlson Index Score, mean (SD)	5.3 (2.3)	4.6 (2)	5.8 (2.1)	6.1 (2.7)	0.018
COTE Index Score, mean (SD)	1.5 (2.4)	1.6 (2.5)	1 (2)	1.8 (2.6)	0.144
BODEx, mean (SD)	2.2 (1.8)	3 (1.8)	1.3 (1.3)	1.7 (1.5)	<0.001
Exacerbations last year, median (IQR) - Moderate exacerbations - Severe exacerbations	1 (0; 2) 1 (0; 2) 0 (0; 1)	2 (1; 2) 1 (0; 2) 0 (0; 1)	0 (0; 1) 0 (0; 1) 0 (0; 0)	1 (0; 2) 0 (0; 1) 0 (0; 1)	<0.001 0.003 0.163
mMRC dyspnoea, median (IQR)	1 (1; 2)	2 (1; 2)	0.8 (0.8)	1.3 (0.7)	<0.001
Minutes walked/day, mean (SD)	75 (85.6)	74.6 (120.7)	84.5 (45.3)	63.8 (36.6)	0.069
Postbronchodilator FEV1 (mL) Postbronchodilator FEV1 (%) Postbronchodilator FVC (mL) Postbronchodilator FVC (%) Postbronchodilator FEV1/FVC (%) Positive bronchodilator test, n (%)	1704 (643) 59.7 (20.5) 3117 (921) 86 (20) 54 (11.2) 59 (43.7)	1566 (678) 55 (20.2) 2930 (1114) 81 (21.5) 52 (12.4) 28 (44.4)	1778 (583) 64.6 (19.6) 3182 (881) 88.8 (20.5) 53.7 (11.2) 19 (52.8)	1862 (625) 61.5 (21.3) 3194 (833) 85.6 (19.1) 57 (8.5) 12 (33.3)	0.047 0.035 0.381 0.117 0.032 0.248
6MWD, metres, mean (SD)	410 (120)	397 (115)	453 (90)	396.4 (145)	0.120
Blood eosinophils, mean (SD) CRP, mean (SD) Fibrinogen, mean (SD)	317.7 (270) 3.4 (6.1) 392 (573)	280.9 (230) 3.7 (7.5) 442 (822)	336 (327.8) 3.5 (4.6) 332 (179)	371.3 (277) 2.9 (4.3) 363 (153)	0.277 0.783 0.450
LAMA LABA/ICS LABA/LAMA LABA/LAMA/ICS Montelukast Azithromycin Mucolytics	3 (1.9) 27 (17.4) 23 (14.8) 101 (65.2) 20 (12.7) 10 (6.4) 7 (4.5)	0 8 (10.4) 7 (9.1) 62 (80.5) 10 (12.8) 9 (11.5) 4 (5.1)	2 (5.1) 9 (23.1) 7 (17.9) 20 (51.3) 7 (17.9) 1 (2.6) 3 (7.7)	1 (2.6) 10 (25.6) 9 (23.1) 19 (48.7) 3 (7.5) 0 0	0.158 0.069 0.111 <0.001 0.379 0.028 0.234
EQ-5D EVA, mean (SD)	61.9 (20.4)	53.1 (19.6)	74.1 (16.4)	67.2 (17.7)	<0.001
EQ-5D index, mean (SD)	0.8 (0.2)	0.7 (0.2)	0.9 (0.15)	0.8 (0.2)	<0.001
HADS—Anxiety, mean (SD)	5.8 (4.5)	7.8 (4.6)	3.2 (3.5)	4.4 (2.9)	<0.001
HADS—Depression, mean (SD)	4.6 (4.5)	6.9 (4.9)	1.9 (2.5)	2.8 (2.9)	<0.001
Total HADS, mean (SD)	10.4 (8.5)	14.6 (9)	5.2 (5.3)	7.2 (5)	<0.001
CAT score, mean (SD)	14.5 (8.7)	20.9 (7)	5.4 (2.7)	11.1 (4.6)	<0.001
ACT score, mean (SD)	17.9 (5.2)	13.8 (3.6)	23.1 (1.8)	20.6 (3.6)	<0.001

Abbreviations: BMI, body mass index; COTE, COPD specific comorbidity test; BODEx, body mass index, obstruction, dyspnoea and history of severe exacerbation; mMRC, modified Medical Research Council dyspnoea scale; 6MWD, 6-min walking distance.; FEV1, forced expiratory volume in 1 s; FVC, forced vital capacity; CRP, C-reactive protein; EQ-5D, European Quality of Life—5 Dimensions; LAMA, long-acting anticholinergic; LABA: long-acting beta-2 adrenergic; ICS: inhaled corticosteroid; HADS, Hospital Anxiety and Depression Scale; CAT, COPD Assessment Test; ACT, Asthma Control Test. *p*-values are the results of the Student *t*-test or ANOVA tests with the Bonferroni correction for multiple comparisons.

**Table 3:** Correlations between the CAT and ACT scores and other variables of interest.

	CAT r Spearman (*p*-Value)	ACT r Spearman (*p*-Value)
Total number of exacerbations	0.237 (0.003)	−0.337 (<0.001)
Total number of moderate exacerbations	0.192 (0.016)	−0.277 (<0.001)
Total number of severe exacerbations	0.179 (0.026)	−0.249 (0.002)
BODEx	0.387 (<0.001)	−0.484 (<0.001)
Postbronchodilator FVC (% pred)	−0.141 (0.086)	0.127 (0.121)
Postbronchodilator FEV1 (% pred)	−0.206 (0.011)	0.234 (0.004)
Postbronchodilator FEV1/FVC (%)	−0.123 (0.134)	0.169 (0.039)
Dyspnoea (mMRC)	0.539 (<0.001)	−0.562 (<0.001)
Metres walked (6MWD)	−0.122 (0.171)	0.174 (0.050)
EQ-5D EVA, mean (SD)	−0.410 (*p* < 0.001)	0.526 (*p* < 0.001)
EQ-5D index, mean (SD)	−0.491 (*p* < 0.001)	0.524 (*p* <0.001)
HADS—Anxiety, mean (SD)	0.514 (*p* < 0.001)	−0.535 (*p* < 0.001)
HADS—Depression, mean (SD)	0.572 (<0.001)	−0.508 (*p* < 0.001)
Total HADS, mean (SD)	0.573 (*p* < 0.001)	−0.564 (*p* < 0.001)

Abbreviations: BODEx, body mass index, obstruction, dyspnoea and history of severe exacerbation; mMRC, modified Medical Research Council dyspnoea scale; 6MWD, 6-min walking distance.; FEV1, forced expiratory volume in 1 s; FVC, forced vital capacity; EQ-5D, European Quality of Life—5 Dimensions; HADS, Hospital Anxiety and Depression Scale; CAT, COPD Assessment Test; ACT, Asthma Control Test.

**Table 4:** Simple and multiple linear regression analyses to predict ACT and CAT scores.

ACT						
	Simple			Multiple		
Variables	Beta	95% CI	*p*-Value	Beta	95% CI	*p*-Value
Age	0.035	−0.056 to 0.127	0.446			
BMI	−0.014	−0.168 to 0.140	0.857			
EQ-5D index	13.172	10.134 to 16.211	<0.001	5.316	2.058 to 8.575	0.002
HADS total	−0.349	−0.430 to −0.269	<0.001	−0.188	−0.270 to −0.106	<0.001
Total Charlson Index Score	0.356	−0.007 to 0.720	0.055			
Metres walked (6MWD)	0.003	−0.007 to 0.013	0.596			
Number of exacerbations	−1.045	−1.519 to −0.571	<0.001	−0.614	−1.035 to −0.193	0.005
Dyspnoea (mMRC)	−3.066	−3.782 to −2.351	<0.001	−2.784	−3.509 to −2.058	<0.001
Postbronchodilator FEV1 (%)	0.058	0.019 to 0.097	0.004			
Sex, male	−0.744	−2.531 to 1.042	0.412			
Smoking	−0.179	−1.980 to 1.621	0.844			
**CAT**						
	**Simple**			**Multiple**		
**Variables**	**Beta**	**95% CI**	***p*** **-Value**	**Beta**	**95% CI**	***p*** **-Value**
Age	−0.140	−0.292 to 0.012	0.071	−0.140	−0.267 to −0.012	0.033
BMI	0.122	−0.134 to 0.378	0.348			
EQ-5D index	−18.831	−24.227 to −13.434	<0.001			
HADS total	0.660	0.535 to 0.786	<0.001	0.527	0.404 to 0.650	<0.001
Total Charlson Index Score	−0.814	−1.415 to −0.212	0.008			
Metres walked (6MWD)	0.004	−0.012 to 0.021	0.612			
Number of exacerbations	1.247	0.431 to 2.064	0.003			
Dyspnoea (mMRC)	4.920	3.701 to 6.138	<0.001	4.919	3.715 to 6.124	<0.001
Postbronchodilator FEV1(%)	−0.082	−0.147 to −0.016	0.015			
Sex, male	0.880	−2.112 to 3.871	0.562			
Smoking	0.360	−2.652 to 3.372	0.814			

Abbreviations: BMI, body mass index; mMRC, modified Medical Research Council dyspnoea scale; 6MWD, 6-min walking distance; FEV1, forced expiratory volume in 1 s; EQ-5D, European Quality of Life—5 Dimensions; HADS, Hospital Anxiety and Depression Scale; CAT, COPD Assessment Test; ACT, Asthma Control Test. *p*-values as results of the Student *t*-test or ANOVA tests with Bonferroni correction for multiple comparisons.

**Table 5:** Characteristics of discordant patients: patients with good CAT but poor ACT scores (CAT < 10 and ACT < 20) and patients with poor CAT and good ACT scores (CAT > 10 and ACT ≥ 20).

Variables	Good CAT (<10) and Poor ACT (<20) (n = 11)	Poor CAT (>10) and Good ACT (≥20) (n = 28)	*p*-Value
Age, mean (SD)	70.4 (8.8)	63.6 (10.2)	0.078
Sex, (male), n (%)	8 (72.7)	22 (78.6)	0.679
Smoker, n (%) Ex-smoker, n (%)	3 (27.3) 8 (72.7)	8 (28.6) 20 (71.4)	0.935
Smoking pack-years, mean (SD)	40.2 (26.3)	42.3 (24.5)	0.541
BMI, mean (SD)	23.9 (4.7)	28.1 (4.8)	0.031
Total Charlson Index Score, mean (SD)	6.8 (3.2)	5.9 (2.6)	0.533
COTE Index Score, mean (SD)	2.7 (2.1)	1.4 (2.7)	0.014
BODEx, mean (SD)	2 (1.7)	1.6 (1.4)	0.554
Total exacerbations last year, median (IQR) - Moderate exacerbations - Severe exacerbations	1 (0; 2) 1 (0; 1) 0 (0; 1)	0.5 (0; 2) 0 (0;1.5) 0 (0; 0)	0.676 0.595 0.436
mMRC dyspnoea, mean (SD)	1.5 (0.5)	1.3 (0.8)	0.390
Minutes walked/day, mean (SD)	67.9 (35.8)	62.6 (37.5)	0.652
FEV1 (mL) FEV1 (%) FVC (mL) FVC (%) FEV1/FVC (%) Positive bronchodilator test	1694 (616) 60.4 (16.7) 3121 (763) 92.9 (16.1) 51 (8.9) 6 (60)	1922.5 (628) 61.9 (23.1) 3220.4 (868) 82.9 (19.7) 59.2 (7.4) 6 (23.1)	0.075 0.090 0.273 0.894 0.015 0.035
6MWD, metres, mean (SD)	359 (154)	409 (143)	0.509
Blood eosinophil count, mean (SD) CRP, mean (SD) Fibrinogen, mean (SD)	392 (233) 2.9 (2.9) 438 (115)	363 (296) 3 (4.7) 336 (158)	0.294 0.571 0.124
EQ-5D EVA, mean (SD)	56.6 (20.3)	71.4 (15)	0.042
EQ-5D index, mean (SD)	0.8 (0.1)	0.8 (0.2)	0.504
HADS score—anxiety, mean (SD)	5.1 (2.5)	4.1 (3.1)	0.086
HADS score—depression, mean (SD)	2.6 (2.5)	2.9 (3.1)	0.352
Total HADS score, mean (SD)	7.6 (4.1)	7.1 (5.3)	0.480
CAT sore	6.1 (2.8)	13.1 (3.5)	<0.001
ACT score	16.6 (3)	22.2 (1.8)	<0.001

Abbreviations: BMI, body mass index; COTE, COPD specific comorbidity test; BODEx, body mass index, obstruction, dyspnoea and history of severe exacerbation; mMRC, modified Medical Research Council dyspnoea scale; 6MWD, 6-min walking distance.; FEV1, forced expiratory volume in 1 s; FVC, forced vital capacity; CRP, C-reactive protein; EQ-5D, European Quality of Life—5 Dimensions; HADS, Hospital Anxiety and Depression Scale; CAT, COPD Assessment Test; ACT, Asthma Control Test. *p*-values as results of the Student *t*-test or ANOVA tests with Bonferroni correction for multiple comparisons.

## Data Availability

The data presented in this study are available on request from the corresponding author.
